# Optimal regimen of trastuzumab in combination with oxaliplatin/ capecitabine in first-line treatment of HER2-positive advanced gastric cancer (CGOG1001): a multicenter, phase II trial

**DOI:** 10.1186/s12885-016-2092-9

**Published:** 2016-02-08

**Authors:** Jifang Gong, Tianshu Liu, Qingxia Fan, Li Bai, Feng Bi, Shukui Qin, Jinwan Wang, Nong Xu, Ying Cheng, Yuxian Bai, Wei Liu, Liwei Wang, Lin Shen

**Affiliations:** Key laboratory of Carcinogenesis and Translational Research (Ministry of Education/Beijing), Department of Gastrointestinal Oncology, Peking University Cancer Hospital and Institute, #52 Fucheng Road, Haidian District, Beijing, 100142, P. R. China; Zhongshan Hospital, Fudan University, Shanghai, China; The First Affiliated Hospital of Zhengzhou University, Zhengzhou, China; General Hospital of the Chinese People’s Liberation Army, Beijing, China; West China Medical School, West China Hospital, Sichuan University, Chengdu, Sichuan, China; Cancer Center of People’s Liberation Army, 81 Hospital of People’s Liberation Army, Nanjing, China; Cancer Hospital/Institute, Chinese Academy of Medical Sciences, Beijing, China; The First Affiliated Hospital of Medical School of Zhejiang University, Hangzhou, Zhejiang China; Jilin Provincial Cancer Hospital, Changchun, China; Cancer Hospital, Harbin Medical University, Harbin, China; Hebei Medical University Fourth Hospital, Hebei Provincial Tumor Hospital, Shijiazhuang, Hebei China; Shanghai First People’s Hospital, Shanghai, China

**Keywords:** Advanced gastric cancer, HER2-positive, Oxaliplatin, Trastuzumab, Capecitabine

## Abstract

**Background:**

The ToGA study showed that trastuzumab plus chemotherapy prolonged median survival in patients with human epidermal growth factor receptor 2 (HER2)-positive advanced gastric cancer. Among chemotherapy options, oxaliplatin might be as effective as cisplatin but has shown to be more tolerable. To further improve treatment options for patients with advanced gastric cancer, we initiated a study to evaluate the efficacy and safety of trastuzumab plus oxaliplatin/capecitabine in patients with HER2-positive advanced gastric cancer.

**Methods:**

CGOG1001 was an open-label, multicenter, prospective phase II study. Patients with chemotherapy-naive HER2-positive advanced gastric cancer were eligible. Trastuzumab was administered at a loading dose of 8 mg/kg followed by 6 mg/kg infusion every 3 weeks (q3w). Oxaliplatin was administrated as a 130 mg/m^2^ infusion, q3w, for up to 6 cycles. Capecitabine 1000 mg/m^2^ was given orally twice daily on days 1–14 followed by a 7-day rest interval. Trastuzumab and capecitabine were continued until disease progression or intolerable toxicity. The primary endpoint was objective response rate. Simon two-stage design (H_0_ = 40 %, H_1_ = 60 %, α = 0.05, β = 0.2) by Response Evaluation Criteria In Solid Tumors 1.0 was applied.

**Results:**

Fifty-one patients were enrolled. Confirmed response was recorded in 46 patients. One patient achieved complete response and 33 patients achieved partial response (response rate 34/51 [66.7 %] in the intent-to-treat population). Median follow-up time was 28.6 months, with a median progression-free survival of 9.2 months (95 % confidence interval [CI]: 6.5–11.6) and a median overall survival (OS) of 19.5 months (95 % CI: 15.5–26.0). Patients with a HER2/CEP17 ratio of greater than five achieved improved OS (20.9 vs 19.5 months, *p* = 0.001). The most common adverse events of grade 3 or above were thrombocytopenia (21.6 %), neutropenia (13.7 %), anemia (5.9 %) and leucopenia (3.9 %).

**Conclusion:**

The addition of trastuzumab to oxaliplatin/capecitabine was well tolerated and the results demonstrated encouraging efficacy.

**Trial registration:**

ClinicalTrials.gov NCT01364493.

## Background

The ToGA trial was a phase III, open-label, randomized controlled trial that established that patients with human epidermal growth factor receptor 2 (HER2)-positive advanced gastric or gastroesophageal cancer who received trastuzumab plus capecitabine (a fluoropyrimidine) and cisplatin had better outcomes compared with patients who received chemotherapy alone [1]. Median progression-free survival (PFS) in the trastuzumab plus chemotherapy group was 6.7 months compared with 5.5 months in the chemotherapy-only group. Median overall survival (OS) was also improved in the trastuzumab group (13.8 vs 11.1 months in the chemotherapy-only group). An exploratory analysis of patients whose tumors had high levels of HER2 protein demonstrated a median OS of 16 months for those treated with trastuzumab plus chemotherapy versus 11.8 months for those treated with chemotherapy alone.

In clinical practice, the use of cisplatin requires hydration, and impacts the kidney and gastrointestinal tract, making tolerability particularly challenging for gastric cancer patients. The REAL2 trial demonstrated that oxaliplatin has at least equivalent efficacy and is better tolerated than cisplatin in advanced gastric cancer [2]. A meta-analysis of the ML17032 and REAL2 trials demonstrated that capecitabine-based therapy had superior OS benefits compared with 5-FU combinations in patients with advanced gastroesophageal cancer [3, 4]. In the ToGA trial, the majority of patients received capecitabine rather than 5-FU [1]. Further, capecitabine is administered orally and thus, more convenient than intravenous 5-FU. According to resource-stratified guidelines for the management of gastric cancer from the Asian Oncology Summit 2013, capecitabine/oxaliplatin is a preferred first-line treatment for advanced gastric cancer in Eastern Asian countries [5]. Jing et al. demonstrated that adding trastuzumab to oxaliplatin increases antitumor effect *in vitro* [6]. However, there are no established positive results for this doublet regimen combined with trastuzumab in advanced gastric cancer. Therefore, the Chinese Gastrointestinal Oncology Group (CGOG) initiated this multicenter, phase II, prospective clinical trial (CGOG1001) to evaluate the efficacy and safety of trastuzumab plus oxaliplatin/capecitabine as first-line treatment for HER2-positive advanced gastric cancer.

## Methods

### Patient eligibility

Patients with previously untreated, HER2-positive (immunohistochemical [IHC] 2+/dual silver *in situ* hybridization [DSISH]-positive, or IHC 3+, assessed by the central laboratory in Fudan University Shanghai Cancer Center), histologically confirmed adenocarcinoma of the stomach or gastroesophageal junction with metastatic/irresectable lesion(s) were eligible. Patients with measurable disease according to the Response Evaluation Criteria In Solid Tumors (RECIST) 1.0, age ≥18 years, Eastern Cooperative Oncology Group (ECOG) score 0–2, and adequate organ function of bone marrow, liver and kidney were included. Enrolled patients had to be capable of taking oral medication and had to be fully recovered from any surgery (excluding diagnostic biopsy) performed within 4 weeks prior to study entry. Patients were excluded if they had received previous oxaliplatin or previous systemic therapy for advanced disease (except adjuvant/neoadjuvant chemotherapy completed at least 6 months before enrollment). Patients with heart failure, coronary artery disease or myocardial infarction within the previous 6 months (baseline left ventricular ejection fraction [LVEF] <50 % measured by echocardiography), other previous malignancy within 5 years (except non-melanoma skin cancer) and brain metastasis (known or suspected) were also excluded.

All patients completed an informed consent form prior to study entry. The study was approved by the ethics committees of 11 centers (details in the list of ethics committees), and all procedures were in accordance with the ethical standards of the responsible committee on human experimentation (institutional and national) and with the Helsinki Declaration of 1964 and later versions.

### Treatment

Trastuzumab (Herceptin®, Shanghai Roche Pharmaceuticals Limited) was administered at a loading dose of 8 mg/kg followed by 6 mg/kg infusion every 3 weeks (q3w). Oxaliplatin (Eloxatin®, Sanofi Ltd.) was administrated as a 130 mg/m^2^ infusion, q3w, up to 6 cycles. Capecitabine 1000 mg/m^2^ (Xeloda®, Shanghai Roche Pharmaceuticals Limited) was given orally twice daily on days 1–14 followed by a 7-day rest period. Trastuzumab and capecitabine were continued until disease progression or intolerable toxicity. Dose adjustments of chemotherapy and trastuzumab therapy interruption were allowed to manage toxicity. If LVEF dropped by 10 points from baseline and to below 50 %, trastuzumab was withheld and a repeat LVEF assessment performed within approximately 3 weeks. If LVEF did not improve or worsened, or if clinically significant congestive heart failure developed, discontinuation of trastuzumab was strongly considered, unless the benefits for the individual patient were deemed to outweigh the risks.

### Assessments

Tumor response was evaluated according to RECIST 1.0. Baseline overall tumor assessments were made within 21 days prior to treatment start by a suitable reproducible technique (either computed tomography [CT] or magnetic resonance imaging [MRI] scan) for all patients. Follow-up tumor evaluations were performed at 6-weekly intervals (±7 days) during the first year. The interval was extended to 12 weeks during subsequent years. CT or MRI scans of chest, abdomen and pelvis were carried out at baseline and included scans for metastatic lesions. If no lesions were visible in the chest, X-ray was used instead of CT scan on subsequent visits. CT/MRI was used on the abdomen and pelvis at baseline and subsequent visits for all patients. If the patient reported deteriorating symptoms, an assessment was scheduled as soon as possible. Central review of the CT and MRI results was done to ensure response rates were evaluated accurately.

The National Cancer Institute Common Terminology Criteria for Adverse Events version 4.0 was used to evaluate the clinical safety of treatments in this study. Subjects were assessed for adverse events at each clinic visit and for 6 months after the last intake of study drug. Ultrasound was performed every 12 weeks to evaluate the cardiac function. If LVEF decreased ≥10 %, the ultrasound was repeated within 1 month.

### Statistical analysis

CGOG1001 was an open-label, multicenter, prospective phase II study. The primary endpoint was objective response rate (ORR). Secondary endpoints included tolerability, PFS and OS. To limit the sample size for the first stage and at the same time reduce the risk of the non-responders in this trial, we chose Simon two-stage design to evaluate the efficacy of this regimen [7]. Because of a single arm setting, it would be more accurate to measure and compare ORR than PFS. The sample size was determined as follow hypothesis, assuming H_0_ = 0.40 (null hypothesis) and H_1_ = 0.60 (alternative hypothesis) with a significance level of 0.05 and a power of 80 %. The result specified a sample size of 46 patients distributed in two stages. If seven or more confirmed partial responses (PRs) were achieved after the first 16 patients, recruitment was extended up to 46 patients. Assuming a 10 % drop-out rate, a total number of 51 patients were required.

PFS was calculated from the first day of chemotherapy until the date of progressive disease (PD) or date of death, whichever occurred first. The censoring date was the last date of follow-up or last tumor measurement. OS was calculated from the date the patient gave informed consent to date of death or date of last assessment if date of death was unknown. Duration of response (DOR) was defined as the interval between the first confirmed objective response (complete response [CR] or PR) and PD or the last follow-up assessment. PFS, DOR and OS curves were generated using the Kaplan–Meier method. The level of statistical significance was set at *P* < 0.05. SPSS version 16.0 was used for all analyses.

## Results

### Patient demographics and baseline characteristics

**Fig. 1:**
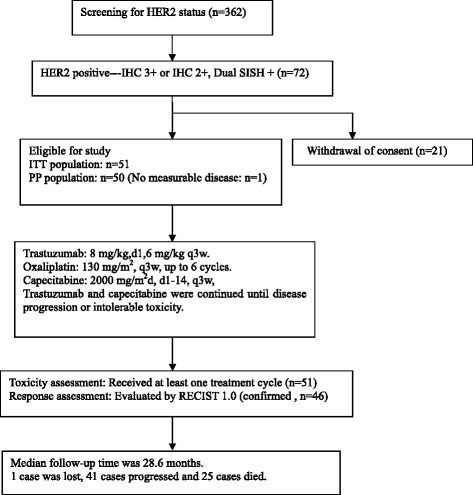
Study flow diagram showing all patients screened for inclusion in the study and reasons for ineligibility

From June 2011 to August 2012, 362 patients from 13 centers were screened. Seventy-two patients were IHC 3+ or IHC 2+/DSISH-positive and 51 patients were enrolled (intent-to-treat [ITT] population). The cut-off date for data analysis was February 28, 2014. Figure 1 shows the reasons for ineligibility and the number of patients included in the analysis. One patient was excluded because of lack of measurable disease according to RECIST 1.0. The median age was 57 years (range 27–78). Thirty-one patients (60.8 %) presented with liver metastases. Thirty-four patients (66.7 %) had intestinal-type disease based on Lauren classification [8]. Patients’ baseline characteristics are shown in Table 1.

**Table 1:** Patients’ characteristics at baseline (*N* = 51)

Patients’ characteristics	Median	%
Median age, years (range)	57 (27–78)	
Sex		
Male	36	70.6
Female	15	29.4
ECOG score		
0-1	48	94.1
2	3	5.9
Primary tumor site		
stomach	33	64.7
Gastroesophageal junction	18	35.3
Type of gastric cancer(assessed by central laboratory)		
Intestinal type	34	66.7
Diffuse type	10	19.6
Mix type	7	13.7
HER2 status		
IHC 3+	38	74.5
IHC 2+,Dual SISH +	13	25.5
HER2/CEP17 ratio by dc-SISH		
2.01–5.0	15	29.4
5.1–50	32	62.7
Failed	4	7.8
Histology		
Well-moderately differentiated	24	47.1
Poorly differentiated (signet ring cell, undetermined)	27	52.9
Extent of disease		
Locally advanced	7	13.7
Metastatic	44	86.3
Metastastic disease		
Liver	31	60.8
Lung	13	25.5
Bone	2	3.9
Ovarian	1	1.9
Lymphnodes	44	86.3
Prior gastrectomy		
No	45	88.2
Yes	6	11.8
Prior adjuvant chemotherapy		
No	50	98.1
Yes	1	1.9

### Efficacy

**Fig. 2:**
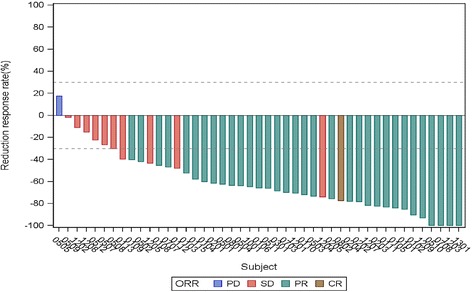
Waterfall plot of overall response of the target lesions measured by RECIST 1.0

**Fig. 3:**
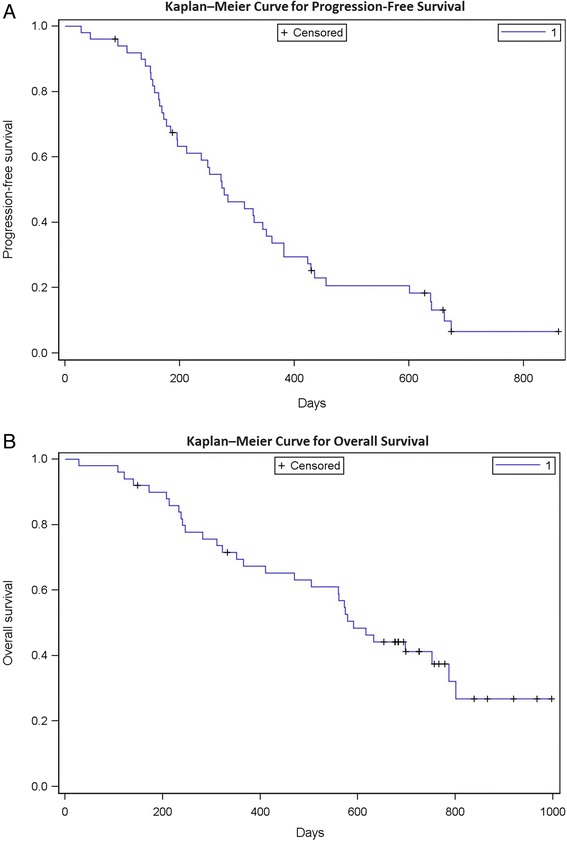
Kaplan–Meier curves for progression-free survival (**a**) and overall survival (**b**)

The median number of treatment cycles given was 8 (range 1–32, total 558 cycles) and the median follow-up time was 28.6 months. Confirmed response was recorded in 46 patients (one CR, 1.9 %; 33 PR, 64.7 %; 10 stable disease, 19.6 %; two PD, 3.9 %; Fig. 2) Two patients had no response evaluation and two patients had early withdrawal of treatment because of serious adverse events. One case was lost to follow-up, 44 cases progressed and 32 cases died by the end of this study. Median PFS was 9.2 months (95 % confidence interval [CI]: 6.5–11.6; Fig. 3) and expected median OS was 19.5 months (95 % CI: 15.5–26.0). DOR was 10.9 months (95 % CI: 8.2–12.6).

We did a further analysis to distinguish the group that would potentially benefit more from trastuzumab plus oxaliplatin/capecitabine. We found a statistically significant correlation between HER2/CEP17 ratio and improved OS. Median HER2/CEP17 ratio was 20 (range 2.01–50.0). Patients who had a HER2/CEP17 ratio of >5 experienced prolonged OS (20.9 versus 19.5 months; *P* = 0.001, data not shown). Subgroup analyses by age, ECOG score, primary disease location, extent of disease, liver metastases and prior gastrectomy were not associated with significant differences in survival.

### Safety

The frequencies of hematological and non-hematological adverse events are shown in Table 2. The most commonly reported adverse events (all grades) were leucopenia (66.7 %), neutropenia (64.7 %), thrombocytopenia (56.9 %), nausea/vomiting (54.9 %) and hepatic dysfunction (45.1 %). The most common grade 3/4 hematological toxicities were thrombocytopenia (21.6 %), neutropenia (13.7 %), anemia (5.9 %) and leucopenia (3.9 %). Nausea/vomiting (3.9 %), diarrhea (3.9 %), hand-foot syndrome (3.9 %) and neurotoxicity (3.9 %) were common grade 3/4 non-hematological toxicities. Eight patients experienced serious adverse events including septic shock, pulmonary tuberculosis, vomiting, upper gastrointestinal hemorrhage, duodenal papillary lesion (not confirmed by pathology), gastroesophageal anastomotic leak, thrombocytopenia and pyloric obstruction. The duodenal papillary lesion was not related to study treatment; therefore, treatment was reintroduced after temporary interruption. Tuberculosis and anastomotic leak were potentially treatment-related and treatment was suspended. Upper gastrointestinal hemorrhage and pyloric obstruction may have been due to PD. Vomiting and thrombocytopenia were potentially related to chemotherapy, leading to early withdrawal of treatment. One patient died of septic shock during the second cycle of chemotherapy. The patient presented at baseline with liver metastases, poor performance status, hypoproteinemia and hypoglycemia.

**Table 2:** Toxicity possibly, probably, or definitely attributable to chemotherapy (*n* = 51)

Adverse events	Grade 1		Grade 2		Grade 3		Grade 4		Total	
	No. of patients	(%)	No. of patients	(%)	No. of patients	(%)	No. of patients	(%)	No. of patients	(%)
Hematological										
Leucopenia	15	29.4	17	33.3	1	1.96	1	1.96	34	66.7
Neutropenia	13	25.5	13	25.5	5	9.8	2	3.9	33	64.7
Anemia	12	23.5	10	19.6	3	5.9	0	0	25	49.0
Thrombocytopenia	7	13.7	11	21.6	7	13.7	4	7.8	29	56.9
Non-hematological										
Hepatic dysfunction	19	37.3	3	5.9	1	1.9	0	0	23	45.1
Nausea/vomiting	15	29.3	11	21.6	2	3.9	0	0	28	54.9
Neurotoxicity	12	23.5	3	5.9	2	3.9	0	0	17	13.7
Hand-food syndrome	12	23.5	5	9.8	2	3.9	0	0	19	37.3
Anorexia	8	15.7	0	0	1	1.9	0	0	9	17.6
Diarrhea	5	9.8	4	7.8	2	3.9	0	0	11	21.6
Infusion reaction	3	5.9	2	3.9	0	0	0	0	5	9.8
Fatigue	1	1.9	3	5.9	1	1.9	0	0	5	9.8
Arrhythmia	2	3.9	3	5.9	0	0	0	0	5	9.8

Six patients experienced asymptomatic LVEF decrease ≥10 % from baseline; five of these had LVEF levels greater than 60 % (three at Weeks 12, 24, and 36, and two during the follow-up period). The sixth patient discontinued trastuzumab due to asymptomatic LVEF below 50 % during Week 12. Trastuzumab was interrupted temporarily in two patients due to arrhythmia during the 13th cycle, but was well tolerated throughout the remainder of the treatment course. No cardiac failure was reported.

The dose of oxaliplatin had to be reduced due to adverse events in 12 patients: nine patients had hematological toxicity (mostly thrombocytopenia and neutropenia) and two patients experienced non-hematological toxicities. The majority of capecitabine dose reductions were due to non-hematological toxicities (15 patients) while two were due to hematological toxicities, namely anemia.

## Discussion

Trastuzumab, an anti-HER2 receptor monoclonal antibody, has emerged as the first targeted drug to improve OS when combined with chemotherapy in advanced HER2-positive gastric cancer [1]. Based on the promising results of the ToGA trial, new, cisplatin-free, less toxic, more convenient, trastuzumab-based first-line regimens are being tested in phase II trials (NCT01359397, NCT01191697, NCT01503983, NCT01228045, NCT01461057) [9]. The results of this study are similar to a study by Ryu et al, which showed that trastuzumab plus oxaliplatin/capecitabine had good efficacy as first-line chemotherapy in HER2-positive advanced gastric cancer [10]. For the ITT population, the ORR observed in our study was 66.7 %, median PFS was 9.2 months and the expected median OS was 19.5 months; these promising results are not inferior to the ToGA trial [1].

Chemotherapy-related toxicities observed in our study were similar to those observed in other clinical trials [1, 2, 11], with thrombocytopenia (21.6 %) and neutropenia (13.7 %) reported as most common grade 3/4 hematological toxicities. Although the most common non-hematological toxicities in our study were hepatic dysfunction and nausea/vomiting, most cases were mild and reversible. Oxaliplatin-related thrombocytopenia and hepatic dysfunction can be managed easily by dose modification. Serious adverse events reported in this trial were slightly higher than Ryu et al reported. The majority of these events were not related to treatment. One patient with poor performance status died of septic shock, which highlights the importance of precautions and active supportive care. The addition of trastuzumab had no impact on chemotherapy tolerability. Although six patients experienced asymptomatic LVEF decrease ≥10 % from baseline on regular surveillance, only one patient discontinued trastuzumab due to LVEF below 50 %. Two patients recovered from arrhythmia after temporary interruption of trastuzumab. Regular monitoring of cardiac function is a vital part of patient care during administration of trastuzumab.

Although trastuzumab-based treatment represents the standard of care for HER2-positive gastric cancer patients, benefits from this regimen may not be as great in certain subgroups of patients. Comprehensive analyses have been conducted to identify patients who will benefit most from anti-HER2 targeted therapy. Gomez-Martin et al. reported the use of HER2 gene amplification level as a predictive factor for response to trastuzumab-based treatment and survival benefit [12]. In that study, 50 patients who had a HER2/CEP17 ratio ≥4.45 survived for more than 12 months. Their median OS was significantly improved compared with patients with a ratio ≤4.45 (median, 21.3 vs. 13.6 months; *P* = 0.005). We conducted an exploratory analysis of HER2/CEP17 ratio to identify patients who would are most likely to benefit from trastuzumab plus oxaliplatin/capecitabine and found that patients with a HER2/CEP17 ratio >5 achieved significantly improved OS than those with HER2/CEP17 ratio ≤5 (20.9 versus 19.5 months; *P* = 0.001). The HER2 amplification level should be considered a significantly predictive biomarker for selecting patients who are most likely to benefit from trastuzumab-based therapy.

At the time of diagnosis, 4–14 % of gastric cancer patients had liver metastases [13–15] and long-term survival is usually hard to achieve in these patients. Li et al. analyzed the survival of 162 Chinese gastric cancer patients with liver metastases who underwent systemic chemotherapy or local treatment [16]. The median OS was 9.5 months. One- and 2-year survival rates were 28.4 and 4.3 %, respectively. Furthermore, in the subgroup analysis of the ToGA trial, patients with visceral (lung or liver) metastases benefitted less from trastuzumab combined with chemotherapy than the overall population [1]. The result of our study showed similar median PFS (278 days) for patients who presented with liver metastases. Therefore, it should be worthwhile to explore the optimal regimen of trastuzumab combined with chemotherapy in gastric cancer patients with synchronous liver metastases. Recently, a randomized, phase III trials (NCT01450696, BO27798) were initiated upon request by the US Food and Drug Administration to evaluate the efficacy of increased dose of trastuzumab for patients with advanced gastric cancer with metastatic disease with documented liver or lung involvement.

Maintenance therapy has shown more benefit compared with discontinuation of chemotherapy in colorectal cancer and lung cancer [17–19]. However, the question remains as to whether this treatment strategy can improve survival for advanced gastric cancer patients. Oncologists’ preferred treatment strategy is to prescribe sufficient chemotherapy to maintain response until progression or intolerance. However, it is challenging to continue combination chemotherapy with good tolerance in clinical practice. Moreover, gastric cancer patients who are diagnosed at the advanced stage generally have poor performance status [5]. In the ToGA trial, trastuzumab was designed to be administered until PD to maximize clinical benefit without negatively influencing quality of life [1]. Based on the published preliminary results of a phase II trial that showed promising efficacy for first-line paclitaxel plus capecitabine in gastric cancer [20], and because of the convenient oral administration of capecitabine, we optimized the regimen in the current study to continue trastuzumab plus capecitabine until PD and demonstrated greater improvement in PFS than that observed in the ToGA trial. We reduced oxaliplatin exploration with fewer grade 3-4 peripheral neuropathy events (4 %) than reported by Ryu et al. (11 %). Therefore, further exploration of this treatment model in advanced gastric cancer patients is worthwhile. On the basis of the CLASSIC trial [11], adjuvant chemotherapy with oxaliplatin/capecitabine is recommended in patients with stage II-III gastric cancer who underwent R0 resection. Our results might provide rationale for further investigations of trastuzumab plus chemotherapy as adjuvant treatment in HER2-positive gastric cancer patients.

## Conclusion

The addition of trastuzumab to oxaliplatin/capecitabine for treatment-naive HER2-positive gastric cancer patients was well tolerated and the results demonstrated encouraging efficacy. Further large-scale studies are required to determine survival benefit.

### List of ethics committees

This study was approved by the ethics committee of Peking University Cancer Hospital; the ethics committee of Zhongshan Hospital; the ethics committee of The First Affiliated Hospital of Zhengzhou University; the ethics committee of General Hospital of the Chinese People’s Liberation Army; the ethics committee of West China Hospital, 81 Hospital of People’s Liberation Army; the ethics committee of Cancer Hospital Chinese Academy of Medical Sciences; the ethics committee of The First Affiliated Hospital of Medical School of Zhejiang University; the ethics committee of Jilin Provincial Cancer Hospital; the ethics committee of Cancer Hospital Harbin Medical University; the ethics committee of Hebei Medical University Fourth Hospital; the ethics committee of Shanghai First People’s Hospital.

## Abbreviations

CGOG: Chinese Gastrointestinal Oncology Group
CI: confidence interval
CR: complete response
CT: computed tomography
DOR: duration of response
DSISH: dual silver *in situ* hybridization
ECOG: Eastern Cooperative Oncology Group
HER2: human epidermal growth factor receptor 2
IHC: immunohistochemical
ITT: intent-to-treat
LVEF: left ventricular ejection fraction
MRI: magnetic resonance imaging
ORR: objective response rate
OS: overall survival
PD: progressive disease
PFS: progression-free survival
PR: partial response
q3w: every three weeks
RECIST: Response Evaluation Criteria In Solid Tumors
SD: stable disease
